# 25-Hydroxyvitamin D in pregnancy and genome wide cord blood DNA methylation in two pregnancy cohorts (MoBa and ALSPAC)

**DOI:** 10.1016/j.jsbmb.2016.03.005

**Published:** 2016-05

**Authors:** M. Suderman, L.C. Stene, J. Bohlin, C.M. Page, K. Holvik, C.L. Parr, M.C. Magnus, S.E. Håberg, B.R. Joubert, M.C. Wu, S.J. London, C. Relton, W. Nystad

**Affiliations:** a MRC Integrative Epidemiology Unit, University of Bristol, Oakfield House, Oakfield Grove, Clifton, Bristol BS8 2BN, UK; b Norwegian Institute of Public Health, Division of Epidemiology, Marcus Thranes Gate 6, P.O. Box 4404, 0403 Oslo, Norway; c Norwegian Institute of Public Health, Infection Control and Environmental Health, Lovisenbergata 8, P.O. Box 4404, 0403 Oslo, Norway; d National Institute of Environmental Health Sciences, National Institutes of Health, Dept. of Health and Human Services, ​P.O. Box 12233, MD A3-05, Research Triangle Park, NC 27709, United States; e Public Health Sciences Division, Fred Hutchinson Cancer Research Center, Seattle, WA 98109, United States

**Keywords:** DNA methylation, Epigenetics, Vitamin D, 2-Hydroxyvitamin D, Maternal vitamin D, Methylome, Offspring

## Abstract

The aim of the study was to investigate whether maternal mid-pregnancy 25-hydroxyvitamin D concentrations are associated with cord blood DNA methylation.

DNA methylation was assessed using the Illumina HumanMethylation450 BeadChip, and maternal plasma 25-hydroxyvitamin D was measured in 819 mothers/newborn pairs participating in the Norwegian Mother and Child Cohort (MoBa) and 597 mothers/newborn pairs participating in the Avon Longitudinal Study of Parents and Children (ALSPAC).

Across 473,731CpG DNA methylation sites in cord blood DNA, none were strongly associated with maternal 25-hydroxyvitamin D after adjusting for multiple tests (false discovery rate (FDR) > 0.5; 473,731 tests). A meta-analysis of the results from both cohorts, using the Fisher method for combining p-values, also did not strengthen findings (FDR > 0.2). Further exploration of a set of CpG sites in the proximity of four *a priori* defined candidate genes (*CYP24A1*, *CYP27B1*, *CYP27A1* and *CYP2R1*) did not result in any associations with FDR < 0.05 (56 tests). In this large genome wide assessment of the potential influence of maternal vitamin D status on DNA methylation, we did not find any convincing associations in 1416 newborns. If true associations do exist, their identification might require much larger consortium studies, expanded genomic coverage, investigation of alternative cell types or measurements of 25-hydroxyvitamin D at different gestational time points.

## Highlights

• Pregnancy vitamin D and cord blood DNA methylation compared in 1416 infants.
• Associations tested in two large prospective cohort studies, MoBa and ALSPAC.
• Similarly for offspring health outcomes and vitamin D, no convincing associations.

## 1 Introduction

Vitamin D is a precursor of the steroid hormone 1,25-dihydroxyvitamin D (1,25(OH)_2_D), with important roles in calcium and bone metabolism as well as other biological processes. A number of different tissues express the vitamin D receptor (*VDR*), which acts as a transcriptional factor after binding of 1,25(OH)_2_D and heterodimerisation with retinoic X receptor (*RXR*). The major circulating form and indicator of vitamin D status, 25-hydroxyvitamin D (25(OH)D), is supplied by 25-hydroxylation of vitamin D produced in the skin upon UVB radiation or vitamin D from the diet [1]. 25(OH)D is activated in a second hydroxylation step, by 1α-hydroxylase (encoded by *CYP27B1*), primarily in the kidneys, but also in other tissues expressing *CYP27B1* including lymphocytes [1], [2].

Low 25(OH)D levels have been associated with a range of adverse conditions, from pregnancy outcomes to childhood illnesses and chronic disease including osteoporosis, cancer and cardiovascular disease in adulthood [3], although randomized controlled trials of vitamin D supplements do not support causality for extra-skeletal outcomes [4], [5].

Vitamin D metabolism changes during pregnancy, suggesting importance for the mother and fetus [6]. For instance, while circulating 1,25(OH)_2_D is normally tightly controlled by renal hydroxylation of 25(OH)D, levels increase during pregnancy. In addition to increased renal expression of *CYP27B1*, this may also partly result from placental expression of *CYP27B1* combined with reduced activity of *CYP24A1,* which catalyzes the first step of the catabolism of 1,25(OH)_2_D.

Lower maternal 25(OH)D during pregnancy has been associated with a number of adverse perinatal outcomes, such as low birth weight and preterm birth and also later health outcomes in the offspring such as bone health [7], wheezing and atopic disorders [8], and autoimmune disorders like type 1 diabetes [9]. Although a number of studies have reported inverse associations between maternal vitamin D status and postnatal health outcomes, systematic reviews show that there is still substantial heterogeneity between studies in terms of methodology and results, and few or no randomized trials have been performed [6], [8], [10], [11]. Although two recent randomized controlled trials observed suggestive reductions in the incidence of asthma and recurrent wheezing following vitamin D supplementation during pregnancy [12], [13], the primary endpoints were not statistically significant. The evidence for causality of these associations therefore remains largely inconclusive.

In addition to a need for large randomized studies in this field, there is also a need to explore potential mechanisms involved in the hypothesized links between maternal vitamin D status and offspring health. It is well established that maternal 25(OH)D during pregnancy is correlated with cord blood 25(OH)D, but it is possible that some of the observed associations with long term health outcomes may be mediated by fetal programming mechanisms such as DNA methylation in the fetal genome [14]. The activated vitamin D receptor has a large number of potential target genes, identified both experimentally using *in vitro* models and *in silico* by identification of vitamin D responsive elements [15]. Potential target genes included some well-established candidates, such as *CYP27B1* and *CYP24A1*, and a large number of yet unconfirmed genes.

A few smaller studies have investigated DNA methylation at some candidate loci in relation to vitamin D [16]. One recent study examined the association between vitamin D deficiency and genome wide DNA methylation in African children [17]. Another study explored epigenetic regulation of vitamin D converting enzymes [18], while a third found a relationship between methylation of the genes *CYP24R1* and *CYP27A1* and variations in circulating 25(OH)D levels [19]. Epigenome wide studies, in general, are gaining in popularity with the use of the Illumina HumanMethylation450 BeadChip. We are not aware of any published studies on the association between maternal 25(OH)D and genome wide DNA methylation in cord blood. We assessed this association using the Illumina HumanMethylation450 assay in 1416 newborns from two large pregnancy cohort studies: The Norwegian Mother and Child Cohort Study (MoBa) [20] and the Avon Longitudinal Study of Parents and Children (ALSPAC) from the UK [21].

## 2 Materials and methods

### 2.1 The Norwegian Mother and Child Cohort Study (MoBa)

Study population and sample acquisition. The Norwegian Mother and Child Cohort Study (MoBa) is a population-based pregnancy cohort administered by the Norwegian Institute of Public Health (NIPH) [20], [22], [23].

Pregnant women were recruited between 1999 and 2008 from 50 of the 52 hospitals in Norway when attending the routine ultrasound examination at approximately 18 weeks of gestation (98% coverage of all pregnant women). The overall participation rate for MoBa was 41% [20]. Blood samples were drawn from the mother and blood from the umbilical cord vein was collected with a syringe. The handling and quality assurance of the biological material has been thoroughly described previously [24].

The present study is based on data from MoBa version VI (108,863 children in total) with linkage to the Medical Birth Registry of Norway (MBR). Participants constituted two subgroups. The first included a sample among those born between July 2002 and December 2003 with completed questionnaires at 18–22 weeks gestation (n = 17,005 eligible children). The second subgroup included children born between July 2002 and July 2004 with completed questionnaires up to 36 months who were classified as having asthma at 36 months. From these two groups, there were 819 children with maternal 25-hydroxyvitamin D levels and cord blood DNA methylation profiles [25]. The study was approved by the Norwegian Data Inspectorate and the Regional Ethics Committee for Medical Research.

**25-Hydroxyvitamin D levels**. Maternal plasma levels of 25-hydroxyvitamin D_3_ and 25-hydroxyvitamin D_2_ were analyzed at Bevital laboratories (www.Bevital.no), using a liquid chromatography-tandem mass spectrometry method (LC–MS/MS) [25]. The within day coefficient of variance for 25-hydroxyvitamin D_2_ was 4.3–4.5%, while the between day coefficient of variance was 4.6–7.7%. The within day coefficient of variance for 25-hydroxyvitamin D_3_ was 4.4–5.3%, while the between day coefficient of variance was 7.3–8.2%. The contribution of 25-hydroxyvitamin D_2_ in the study sample was negligible, and the sum of 25-hydroxyvitamin D_3_ and -D_2_, termed 25(OH)D, was used in the analysis.

**DNA methylation profile generation**. Cord blood DNA methylation was assayed using the Illumina Infinium HumanMethylation450 BeadChip (www.illumina.com), which was designed to conduct epigenome-wide association studies (EWAS) and includes 485,512 methylation sites per sample at single-nucleotide resolution. This chip covers 99% of RefSeq genes, with an average of 17CpG sites per gene region across the promoter, 5′UTR, first exon, gene body, and 3′UTR. In addition, the chip covers 96% of CpG islands, with additional coverage of island shores. DNA methylation levels of CpG sites is detected using bisulfite-converted genomic DNA (gDNA), where unmethylated (Un) cytosine bases are converted to uracil, while methylated (Me) cytosine remains unchanged. Bisulfite conversion was performed using the EZ-96 DNA Methylation kit (Zymo Research Corporation, Irvine, CA) according to manufacturer instructions. Illumina reports an average Beta value for the methylation level of the interrogated sites based on the following formula: β_ij_ = Max(Me_ij_, 0)/(Max (Me_ij_, 0) + Max (Un_ij_, 0) + 100), for each person *j* and each of the 485,512 CpG sites *i*. Batch effects in these analyses were avoided as all samples were analyzed on the same day, by the same individual, using the same instrument. As previously described, chip, chip set and plate were not appreciable sources of variability [26], [27], so they were not included as covariates in regression models. BMIQ was performed on the methylation data to assimilate type I and II probes [28], [29]. More information regarding quality control of the MoBa cohort dataset can be found elsewhere [26], [27].

**Statistical analyses**. MM type robust linear regression [30] was carried out with CpG values (0 ≤ β ≤ 1) as outcome and plasma 25(OH)D as the explanatory variable. MM type regression is very robust to outliers (breakdown point when 50% incorrect observations compared to 1% for ordinary least squares). It is superior to robust methods using sandwich estimators because it adjusts both coefficient estimates and standard errors rather than just standard errors. The 25(OH)D explanatory variable was approximately normally distributed. All p-values and regression estimates reported are for 25(OH)D in nmol/l as a continuous variable, categorizing the 25(OH)D variable into quartiles made no difference. In the primary analysis, the models were adjusted for maternal pre-pregnancy body mass index (BMI, continuous), offspring sex, maternal education (4 categories), maternal smoking (yes/no), maternal folate plasma values (continuous), parity (3 categories), maternal age (continuous), birth season (four categories) and estimated cell type proportions (continuous matrix consisting of 6 (default) blood cell-types: CD4^+^ T cells, CD8^+^ T cells, NK-cells, B-cells, monocytes and granulocytes). The cell-type estimations were calculated with the minfi package [31], which is based on the method described by Houseman et al. [32]. In a sensitivity analysis, we ran additional models adjusting for various subsets of the covariates mentioned above:

Model 1. None

Model 2. Maternal Age + Folate + Offspring gender + Houseman cell counts

Model 3. Model 2 + Season

Model 4. Model 2 + Maternal education + Maternal smoking + Parity + Maternal BMI

Model 5. Model 4 + Season

We refer to the first as the ‘crude’ model and the last as the ‘full’ model.

We used the genomic control (λ_GC_) [33] to assess model quality and all models tested were found to have λ_GC_ close to one. A λ_GC_ close to one suggests that the assumption of independent and identically distributed tests is fulfilled indicating that the less conservative FDR-based q-values [34] can be used to assess significance in the genome-wide models. Since none of the models produced any associations with FDR < 0.05 and concordant effect sizes, only the results from the crude analysis including no covariates are presented. The meta-analysis was performed using the Fisher method [35] on all the (2×) 473,731 p-values from both MoBa and ALSPAC crude models and FDR adjusted for multiple testing.

### 2.2 The Avon Longitudinal Study of Parents and Children study (ALSPAC)

**Study population and sample acquisition**. This study used DNA methylation data generated under the auspices of the Avon Longitudinal Study of Parents and Children (ALSPAC) [21], [36], [37]. DNA extracted from cord blood and peripheral blood samples along with a wide range of exposure and phenotypic data were used.

**25-Hydroxyvitamin D levels**. Approximately a quarter of the 25(OH)D samples were collected in each of the first two trimesters and half in the third trimester of pregnancy. Because 25(OH)D levels are known to fluctuate during the year (season) and perhaps by gestational week, 25(OH)D were pre-adjusted for season and gestational age at blood sample collection as previously described [38].

**DNA methylation profile generation**. Cord blood DNA methylation was assayed using the Illumina HumanMethylation450 platform and data pre-processed using procedures identical to those used for the MoBa dataset. Bisulfite conversion was performed using the EZ DNA Methylation kit (Zymo Research Corporation, Irvine, CA) according to manufacturer instructions. All steps were performed at the University of Bristol as part of the Accessible Resource for Integrated Epigenomic Studies (ARIES) project (http://www.ariesepigenomics.org.uk). During the data generation process a wide range of batch variables were recorded in a purpose-built laboratory information management system (LIMS). The LIMS also reported QC metrics from the standard control probes on the 450 K BeadChip. Samples failing quality (samples with >20% probes with p-value > = 0.01) were repeated. Samples from all three time points in ARIES were randomized across arrays to minimize the potential for batch effects. As an additional QC step, genotype probes on the 450 K BeadChip were compared between samples from the same individual and against SNP-chip data to identify and remove any sample mismatches. The ALSPAC samples were not analyzed in a single day as were the MoBa samples. Consequently, the dataset was normalized using an alternative approach optimized to minimize the effects of resulting technical artefacts. Specifically, data normalization included background correction and subset quantile normalization using the pipeline described by Touleimat and Tost [39] and implemented in the watermelon R package [29].

### 2.3 Statistical analyses

Associations between DNA methylation and 25(OH)D were tested using procedures and covariate subsets identical to those used for the MoBa study. The only exception was that maternal plasma folate was omitted as it has not been measured in ALSPAC. Due to potentially lingering batch effects present following normalization, additional analyses were performed that included covariates generated using independent surrogate variable analysis (ISVA) [40]. Two versions were considered called ‘isva0’ and ‘isva1’. In ‘isva0’, ISVA was applied to the 25(OH)D levels and DNA methylation data. In ‘isva1’, ISVA was applied as in ‘isva0’ but additionally all covariates, a batch variable (sample plate), and all ‘isva0’ surrogate variables were included as input for generating surrogate variables. The results ‘isva0’/’isva1’ were meta-analyzed with results from the crude/full MoBa models.

## 3 Results

**Fig. 1:**
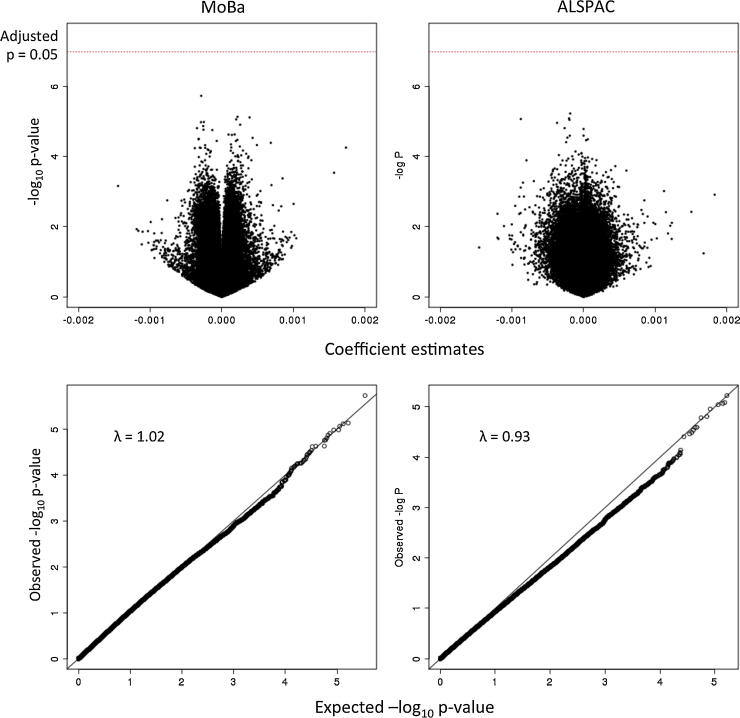
Association statistics consistent with null distributions. Volcano- and qq-plots for regression models having offspring methylation betas as the response variable and maternal vitamin D levels as the explanatory variable for both MoBa (left) and ALSPAC (rights) cohorts.

**Fig. 2:**
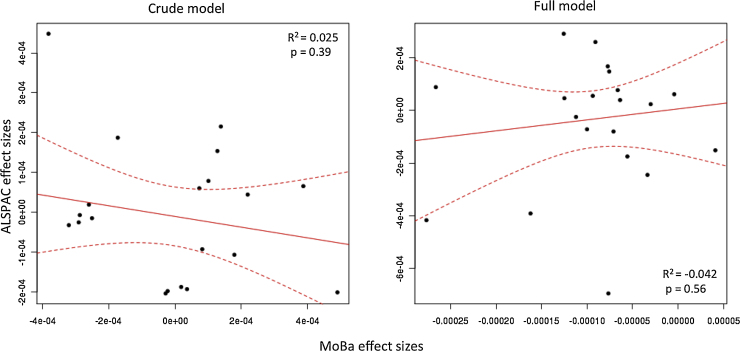
Study effect sizes not correlated between MoBa and ALSPAC. Each scatterplot shows the effect sizes of the top 20 meta-analyzed associations. The left plot shows the effect sizes for the crude model (no covariates), and the right plot shows those for the full model. The dashed lines mark the 95% confidence interval for the regression line.

There was no association between mid-pregnancy 25(OH)D and cord blood DNA methylation at any single site on the Illumina HumanMethylation450 BeadChip among the 819 mother and child-pairs in MoBa and the 597 mother and child-pairs in ALSPAC (at FDR < 0.05; 473,731 tests). Adjustment for potential confounding variables (See Table 1, as well as the Materials and Methods section) and cell-type estimations as well as a meta-analysis, based on Fisher's method, comprising results from both MoBa and ALSPAC cohorts did not result in any association between maternal 25(OH)D levels and DNA methylation in offspring (at FDR < 0.05; 473,731 tests). The 1000 strongest associations are provided in Supplementary Information file 1. Regression estimates tended to be very small, and all p-values were greater than 0.05 after correction for multiple tests (FDR > 0.2, Bonferroni adjusted p > 0.2, 473731 tests). Fig. 1 shows QQ- and volcano plots for both MoBa and ALSPAC cohorts based on coefficient estimates and p-values for 473731CpG probes. Fig. 2 shows the lack of agreement in effect sizes in MoBa and ALSPAC for the top 20 meta-analyzed associations. Information regarding maternal circulating 25(OH)D levels and other covariates used throughout the study can be found in Table 1.

**Fig. 3:**
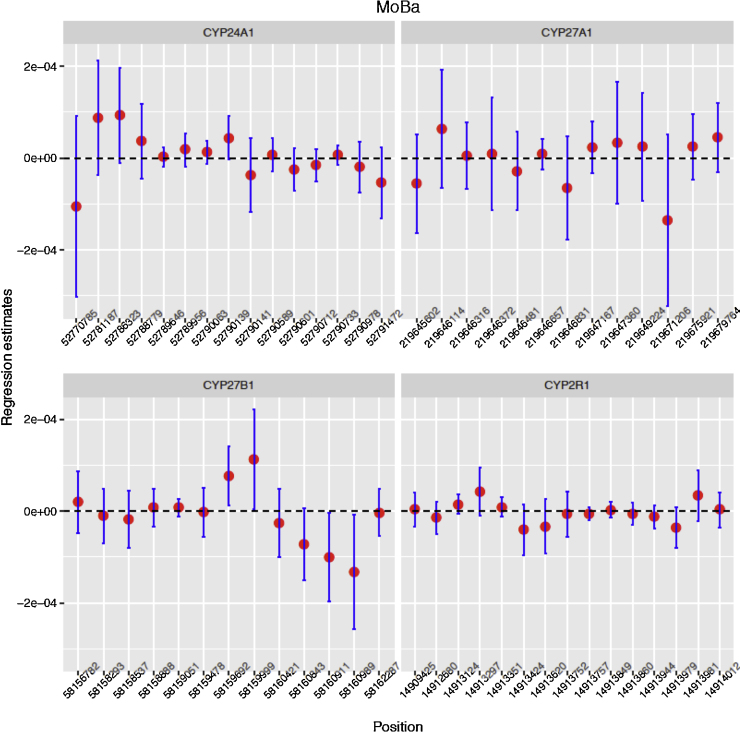
MoBa regression coefficient estimate confidence intervals typically contain zero. Estimated slope between DNA methylation levels of offspring CpG sites and maternal vitamin D levels from the crude model for 4 candidate genes in the MoBa study. The horizontal axis represents the genomic positions of the CpGs, while the vertical axes represent slope estimates. The vertical lines represent ±1.96 × estimated standard error for the regression coefficient.

**Fig. 4:**
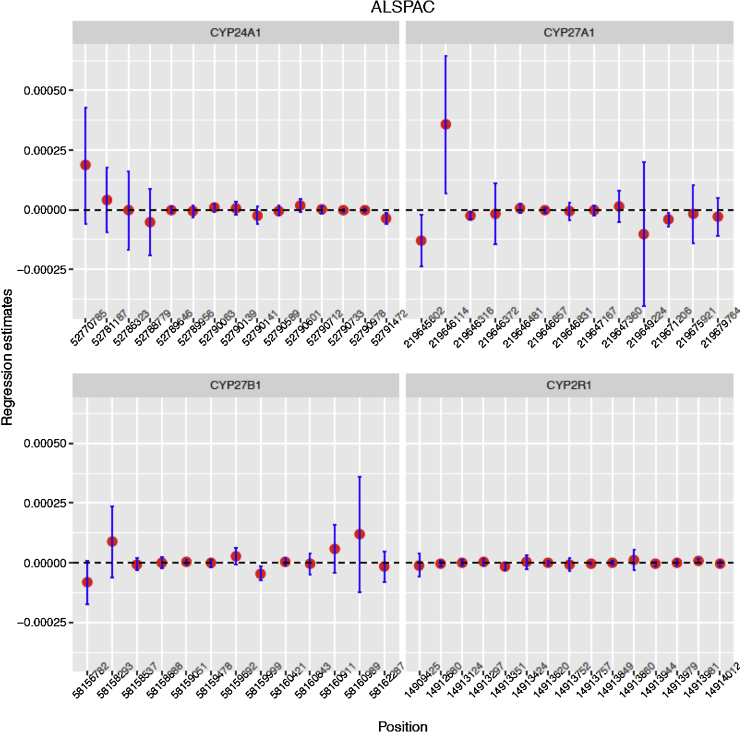
ALSPAC regression coefficient estimate confidence intervals typically contain zero. Estimated slope between methylation levels of offspring CpG sites and maternal vitamin D levels from the crude model for 4 candidate genes in the ALSPAC study. The horizontal axis represents the genomic positions of the CpGs, while the vertical axes represent slope estimates. The vertical lines represent ±1.96 × estimated standard error for the regression coefficient.

Furthermore, detailed analysis of CpG’s linked to the four *a priori* defined candidate genes (*CYP24A1, CYP27B1, CYP27A1* and *CYP2R1*) yielded weak associations with very small regression coefficient estimates (Fig. 3; FDR > 0.6 for 56 tests; see Supplementary information File 2). Repeating the analysis within the ALSPAC cohort, there were similarly only weak associations between maternal 25(OH)D and cord blood DNA methylation among 597 mother and child pairs both at the genome wide and at the four candidate loci (Fig. 4).

## 4 Discussion

We explored the potential influence of maternal mid-pregnancy 25(OH)D on fetal DNA methylation using the Illumina 450 K BeadChip. Despite the existing hypothesis that maternal vitamin D status may influence offspring health [41], we found no evidence for any DNA methylation based effect in cord blood, either genome-wide or in four candidate genes.

The strength of our study is the large sample size from two well-characterized cohorts with 25(OH)D status and genome-wide Illumina HumanMethylation450k data available for a total of 1416 mother and child-pairs. Despite the fact that the Illumina HumanMethylation450 BeadChip only covers 485512CpG sites out of a possible ∼28 million, most of the targeted sites are found in the promoter region [42] which is the predominant region reported to influence gene expression with regards to circulating 25(OH)D levels [19], [43], [44]. Epigenetic effects of maternal vitamin D levels on offspring methylomes can nevertheless not be excluded; the CpG’s in the neighborhood of the candidate genes, as mapped by the Illumina HumanMethylation450 BeadChip, may not be the same as the ones reported from other studies. Although the sample size is relatively large compared to previous DNA methylation studies, it may possibly be too small to detect weak epigenetic effects.

Our analysis in both cohorts was limited to cord blood DNA methylation. It is possible that DNA methylation levels are strongly affected by maternal 25(OH)D levels in some other tissue. Analysis was complicated by the fact that cord blood is composed of several different fluctuating cell types, each with their own distinct DNA methylation profiles. We attempted to control for this by including estimates of cell type proportions in regression models [32]. Although this approach is not ideal, it is currently the only feasible solution [45]. We did not observe strong associations with 25(OH)D using regression models that included nor excluded cell count estimates.

We are not aware of any previously published studies of maternal 25(OH)D and cord blood DNA methylation. However, a few other studies have explored relationships between vitamin D supplementation or circulating 25(OH)D and methylation at a few CpG sites in two to four candidate genes in adults [18], [46], and one genome-wide DNA methylation study explored association with vitamin D deficiency in African children [17].

Given that vitamin D status fluctuates throughout pregnancy, it is possible that the fetus is more sensitive to vitamin D levels at certain gestational periods [6]. In the MoBa study, measurements were taken around 18 weeks gestation, and in the ALSPAC study they were measured throughout pregnancy but normalized to 28 weeks gestation. It is therefore possible that measurements at other time points might have provided stronger associations with cord blood DNA methylation. Hopefully future studies will be designed to systematically investigate timing.

## 5 Conclusions

We found no strong associations between DNA methylation in neonatal genomes and maternal plasma 25(OH)D concentration. Further scrutiny of a set of specific candidate genes did not indicate any association. Our results suggest that similarly powered studies of maternal 25(OH)D in relation to cord blood DNA methylation with the Illumina HumanMethylation450 BeadChip will be unlikely to identify true associations, if they exist. Any future study should utilize DNA methylation profiles of alternative cell types, expanded genomic coverage, larger sample sizes, or measurements of 25(OH)D at different gestational time points.

## Figures and Tables

**Table 1:** Population characteristics.

	MoBa	ALSPAC
Number of participants used	819	597

Maternal age (years)		
Mean ± SD	29.9 (4.33)	30.1 (4.38)

Maternal mid-pregnancy 25-hydroxy vitamin D (ng/mL)		
Mean ± SD	73.5 (23.42)	68.3 (32.3)
Maternal folate levels (nmol/L)		
Mean +/− SD	12.0 (7.99)	N/A

Maternal pre-pregnancy BMI (kg/m^2^)		
Median ± SD	23.2 (4.15)	22.9 (3.71)

Maternal education		
Low	61	52 (CSE)
High school	258	52 (Vocational)
College	367	198 (O level)
University	129	172 (A level)
		114 (Degree)

Maternal smoking during pregnancy		
Yes	110	88
No	702	463

Maternal parity		
First child	346	275
Second child	333	215
Third or more	140	89

Offspring sex		
Male	449	288
Female	370	309

Offspring asthma at 3 yrs		
Yes	328	81 (wheezing)
No	491	516

Birth season		
Feb–Apr	267	121
May–Jul	172	161
Aug–Oct	136	181
Nov–Jan	244	134
